# Scrub typhus and depression: a nationwide cohort analysis

**DOI:** 10.1186/s12967-018-1699-9

**Published:** 2018-12-03

**Authors:** Ying-Chuan Wang, Chun-Hsiang Chiu, Cheng-Li Lin, Feng-You Lee, Kuang-Hsi Chang

**Affiliations:** 1 Department of Family Medicine, Tri-Service General Hospital, National Defense Medical Center, Taipei, Taiwan; 2 Division of Infectious Diseases and Tropical Medicine, Department of Internal Medicine, Tri-Service General Hospital, National Defense Medical Center, Taipei, Taiwan; 3 Institute of Clinical Medicine, School of Medicine, National Yang-Ming University, Taipei, Taiwan; 4 Management Office for Health Data, China Medical University Hospital, Taichung, Taiwan; 5 College of Medicine, China Medical University, Taichung, Taiwan; 6 Department of Emergency Medicine, Taichung Tzu Chi Hospital, Taichung, Taiwan; 7 Department of Medical Research, Tungs’ Taichung Metroharbor Hospital, Taichung, Taiwan; 8 Graduate Institute of Biomedical Sciences, China Medical University, Taichung, Taiwan; 9 General Education Center, Jen-Teh Junior College of Medicine, Nursing and Management, Miaoli, Taiwan

**Keywords:** Scrub typhus, Depression, Risk factor, Endothelial dysfunction, Vascular disease

## Abstract

**Background:**

Studies on the relationship between depression and scrub typhus are limited. We conducted a retrospective cohort study to investigate whether scrub typhus is a risk factor for depression.

**Methods:**

Using Taiwan’s National Health Insurance Research Database, this study investigated the incidence of depression, and its risk factors, in patients diagnosed with scrub typhus between 2000 and 2010. Scrub typhus patients who did not have a history of depression before the index date were enrolled. For each patient with scrub typhus, four controls without a history of scrub typhus and depression were randomly selected and frequency matched by sex, age, year of the index date, and comorbidities. The follow-up period was from the time of initial scrub typhus diagnosis to the date of diagnosis of depression, censoring, or December 31, 2010. Cox proportional hazards regression models were used to analyze the risk of depression according to sex, age, and comorbidities.

**Results:**

The study comprised a 5238-patient scrub typhus group and a 20,952-patient non-scrub typhus group with similar sex and age distributions. During the follow-up period, the cumulative incidence of depression was higher in the scrub typhus than the non-scrub typhus group (log-rank test *P* < 0.001). In the scrub typhus group, 45 patients developed depression, yielding an incidence rate of 1.67 per 1000 person-years, and in the non-scrub typhus group, 117 patients developed depression, yielding an incidence rate of 1.08 per 1000 person-years. This yielded a crude hazard ratio (HR) of 1.55 (95% confidence interval [CI] 1.41–1.70) and adjusted HR (aHR) of 1.56 (95% CI 1.42–1.71). Compared with the non-scrub typhus group, the risk of depression in the scrub typhus group was higher in patients of both sexes (men: aHR = 1.46, 95% CI 1.29–1.64; women: aHR = 1.68, 95% CI 1.45–1.96), in patients aged younger than 65 (≤ 49 years: aHR = 1.95, 50–64 years: aHR = 1.73), and in patients without comorbidities (aHR = 2.06, 95% CI 1.85–2.29).

**Conclusions:**

The risk of depression was 1.56-fold higher in patients with scrub typhus than in the general population.

## Background

Depression is one of the most commonly diagnosed mental disorders among adults and has 12-month and lifetime prevalence rates of 6.6% and 16.2%, respectively [1]. In 2010, depression was the second leading cause of disability as measured using years lived with disability [2]. Depression can be accompanied by several psychophysiological changes such as disturbances in appetite, defecation, and sleep as well as low energy or fatigue. It is also significantly associated with cardiovascular disease, cancer [3], and all-cause mortality [2]. Depression is not a homogeneous disorder but one involving multifactorial etiologies with different subtypes, various courses, and complicated mechanisms.

Scrub typhus (ST) is a mite-borne infectious disease caused by *Orientia tsutsugamushi*, transmitted to humans through the bite of an infected larva (chigger) of a trombiculid mite [4]. It is endemic to the “tsutsugamushi top triangle,” which covers the region encompassed by northern Japan and far-eastern Russia in the north, northern Australia in the south, and Pakistan and Afghanistan in the west [5]. ST may initially present insidiously with headache, anorexia, poor appetite, malaise, chills, and fever [6]. Patients may manifest specific signs such as eschars and nonspecific signs such as lymphadenopathy, skin rash, abnormal liver function, and hepatomegaly. Complications include acute respiratory distress syndrome [7], central nervous system dysfunction [8, 9], acute renal failure [8], and septic shock [9]. Owing to the nonspecific presentations, a delayed diagnosis is not uncommon.

As the severity of the clinical symptoms of ST varies from mild and self-limiting to dysfunction of multiple organs, it is considered vasculotrophic in nature. The current hypothesis regarding immunopathogenic mechanisms implicates oxidative stress, which leads to endothelial cell injury, increased microvascular permeability, and even the development of a hypercoagulable condition [10]. The pathogenesis of endothelial dysfunction may mimic many chronic diseases, including depression. A previous study suggested that ST may increase the risk of developing acute coronary syndrome [11]. Thus, we wondered whether ST may also increase the risk of developing subsequent dementia. This study aims to clarify the risk of depression in patients with ST.

## Methods

### Data source

Patient data for this retrospective population-based cohort study were obtained from the 2000–2011 records of the National Health Insurance Research Database (NHIRD), a database linked to Taiwan’s National Health Insurance (NHI) program. The NHIRD, which has been described in detail elsewhere [11, 12], includes all claim data from the NHI, which covers more than 99% of Taiwan’s population (23.74 million) [13]. The National Health Research Institutes manage the NHIRD, which protects patient privacy by assigning scrambled and random identification numbers to data. In the NHIRD, diagnoses are coded according to the International Classification of Diseases, Ninth Revision, Clinical Modification (ICD-9-CM). This study was approved by the Ethics Review Board of China Medical University and Hospital, Taiwan (CMUH-104-REC2-115).

### Sampled participants

**Fig. 1:**
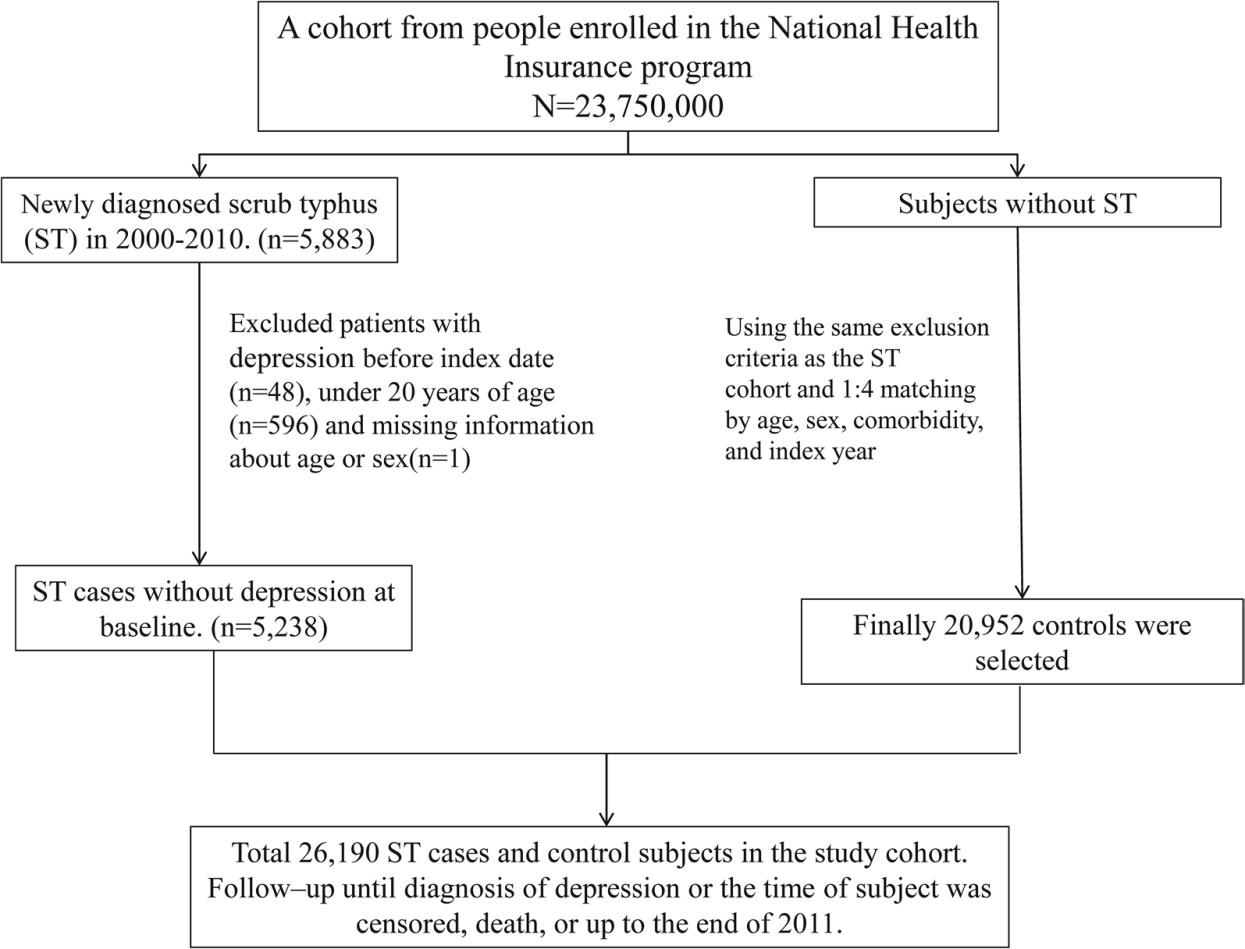
Selection process of the participants in the two study cohorts

The ST group comprised adult patients (aged ≥ 20 years) diagnosed with ST (ICD-9-CM codes 081.0, 081.2, 081.9) between January 1, 2000, and December 31, 2010. The index date was set as the first day of hospitalization of patients diagnosed with ST. To form a comparable non-ST group, we randomly selected individuals without a history of ST from the NHIRD, and they were four-fold frequency matched by age (every 5-year span), sex, and comorbidities (namely diabetes [ICD-9-CM 250], hypertension [ICD-9-CM 401–405], hyperlipidemia [ICD-9-CM 272], coronary artery disease (CAD) [ICD-9-CM 410–414], and stroke [ICD-9-CM 430–438]). We excluded individuals with a history of depression (ICD-9-CM 296.2, 296.3, 296.82, 300.4, 311) before the index date (Fig. 1). Both the ST and non-ST groups were followed from the index date to the date of diagnosis of depression, withdrawal from the insurance program, censoring because of death, or the end date of the database (December 31, 2011).

### Statistical analysis

In our analysis, age and follow-up periods were recorded as means and standard deviations (SDs). Sex, age group, and comorbidities were recorded as absolute numbers and percentages. We computed the cumulative incidences of depression by using the Kaplan–Meier method and estimated the differences in the cumulative incidence curves in the ST and non-ST groups by using the log-rank test. The incidence density rates of depression (per 1000 person-years) were calculated for each risk factor and then stratified by age, sex, and comorbidities. Univariable and multivariable Cox proportional hazard regression models were employed to estimate hazard ratios (HRs) and 95% confidence intervals (CIs) for assessing the effect of ST on the risk of depression. The multivariable Cox models were adjusted for sex, age, and comorbidities, namely diabetes, hypertension, hyperlipidemia, CAD, and stroke. After the patients were stratified according to sex, age, and comorbidities, the risk of depression in the ST group relative to that in the non-ST group was analyzed using Cox models. All analyses were conducted using SAS statistical software (version 9.4 for Windows; SAS Institute Inc., Cary, NC), and all statistical tests were performed at the two-tailed significance level of 0.05.

## Results

### Demographic characteristics, comorbidities, and cumulative incidence of depression in the ST and non-ST groups

The study comprised an ST group of 5238 patients and a non-ST group of 20,952 patients (Table 1). Both groups had similar sex and age distributions, with the majority being women (65.1%) aged 49 years or younger (56.2%). The mean ages for the ST and non-ST groups were 46.6 years (SD = 16.9) and 46.4 years (SD = 16.6), respectively. In both study groups, the major comorbidity was hypertension (13.9%), followed by diabetes (9.97%).

**Table 1:** Demographic characteristics and comorbidity in patient with and without scrub typhus

Variable	Scrub typhus	
	No	Yes
	N = 20,952	N = 5238
Sex	n (%)	n (%)
Female	7316 (34.9)	1829 (34.9)
Male	13,636 (65.1)	3409 (65.1)
Age, mean (SD)	46.4 (17.1)	46.6 (16.9)
Stratify age		
≤ 49	11,772 (56.2)	2943 (56.2)
50–65	5824 (27.8)	1456 (27.8)
65 +	3356 (16.0)	839 (16.0)
Comorbidity		
Diabetes	2088 (9.97)	522 (9.97)
Hypertension	2908 (13.9)	727 (13.9)
Hyperlipidemia	1064 (5.08)	266 (5.08)
CAD	968 (4.62)	242 (4.62)
Stroke	688 (3.28)	172 (3.28)

**Fig. 2:**
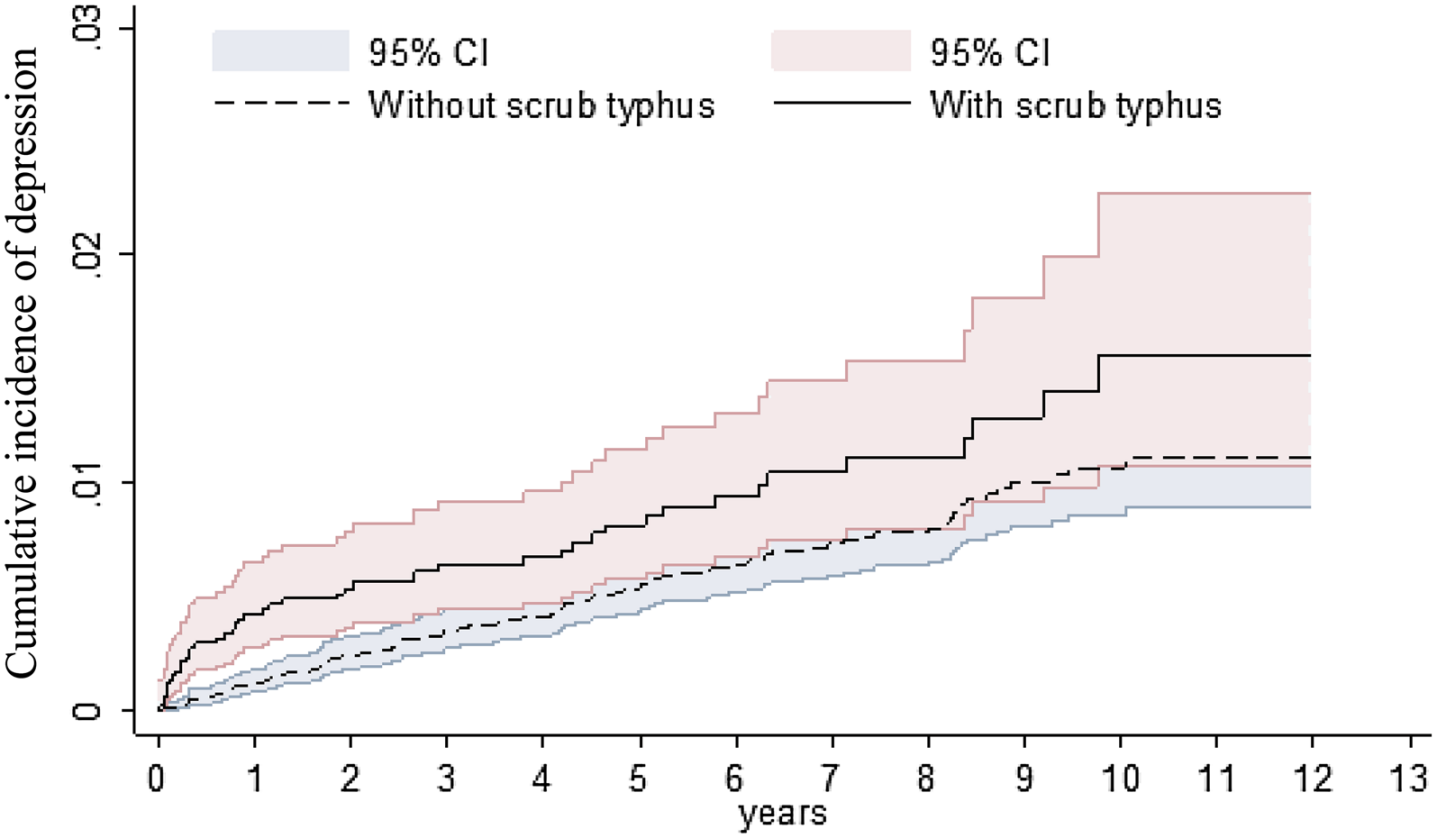
Cumulative incidence of depression compared between with and without Scrub typhus cohorts using the Kaplan-Meier method

The Kaplan–Meier analysis revealed that during the mean follow-up periods (5.14 years and 5.18 years for the ST and non-ST groups, respectively), the ST group exhibited a higher cumulative incidence of depression than did the non-ST group (log-rank test, *P* < 0.001; Fig. 2).

### Incidence and HRs of depression and other risk factors in the ST and non-ST groups

The overall incidences of depression in the ST and non-ST groups were 1.67 and 1.08 per 1000 person-years, respectively (Table 2). After adjustment for sex, age, and comorbidities (namely diabetes, hypertension, hyperlipidemia, CAD, and stroke), the risk of depression was higher in the ST group than in the non-ST group (adjusted HR [aHR] = 1.56, 95% CI 1.42–1.71). In the multivariable model, the risk of depression was 1.35-fold higher in men than in women (95% CI 1.24–1.47). Compared with patients aged 49 years or younger, the risk of depression was 1.26-fold higher in those aged 65 years or older (95% CI 1.11–1.42). The risk of developing depression was higher in patients with comorbidities, namely diabetes (aHR = 1.53, 95% CI 1.35–1.73), hypertension (aHR = 1.41, 95% CI 1.24–1.59), hyperlipidemia (aHR = 2.08, 95% CI 1.81–2.40), CAD (aHR = 1.51, 95% CI 1.29–1.76), and stroke (aHR = 1.52, 95% CI 1.27–1.81).

**Table 2:** Incidence and Hazard ratio for depression and depression-associated risk factor

Variable	Event	Person-years	Rate^a^	Crude HR (95% CI)	Adjusted HR^b^ (95% CI)
Scrub typhus					
No	117	108,433	1.08	1.00	1.00
Yes	45	26,905	1.67	1.55 (1.41, 1.70)*	1.56 (1.42, 1.71)*
Sex					
Female	72	46,692	1.54	1.52 (1.39, 1.65)*	1.35 (1.24, 1.47)*
Male	90	88,645	1.02	1.00	1.00
Age, year					
≤ 49	77	81,148	0.95	1.00	1.00
50–64	50	35,400	1.41	1.49 (1.35, 1.64)*	1.10 (0.99, 1.22)
65 +	35	18,789	1.86	1.96 (1.76, 2.19)*	1.26 (1.11, 1.42)*
Diabetes					
No	133	124,334	1.07	1.00	1.00
Yes	29	11,003	2.64	2.46 (2.21, 2.75)*	1.53 (1.35, 1.73)*
Hypertension					
No	125	120,702	1.04	1.00	1.00
Yes	37	14,636	2.53	2.44 (2.21, 2.70)*	1.41 (1.24, 1.59)*
Hyperlipidemia					
No	141	129,514	1.09	1.00	1.00
Yes	21	5823	3.61	3.31 (2.92, 3.76)*	2.08 (1.81,2 .40)*
CAD					
No	144	130,203	1.11	1.00	1.00
Yes	18	5133	3.51	3.17 (2.77, 3.63)*	1.51 (1.29, 1.76)*
Stroke					
No	150	131,863	1.14	1.00	1.00
Yes	12	3474	3.45	3.04 (2.58, 3.57)*	1.52 (1.27, 1.81)*

^a^Incidence rate, per 1000 person-years; Crude HR, relative hazard ratio

^b^Multivariable analysis including age, sex, and comorbidities of diabetes, hypertension, hyperlipidemia, CAD and stroke

* P < 0.05

### Incidence and HRs of depression stratified by sex, age, and comorbidities in the ST and non-ST groups

Table 3 presents the incidence and HRs of depression in the ST and non-ST groups calculated using Cox models after stratification by age, sex, and comorbidities. The sex-specific relative risk of depression was higher in the ST group than in the non-ST group for both women (aHR = 1.68, 95% CI 1.45–1.96) and men (aHR = 1.46, 95% CI 1.29–1.64) (Table 2). The age-specific relative risk of depression was higher in the ST group than in the non-ST group for patients aged 49 years or younger (aHR = 1.95, 95% CI 1.72–2.20) and those aged 50–64 years (aHR = 1.73, 95% CI 1.46–2.05). The relative risk of depression was higher in the ST group than in the non-ST group for patients without comorbidities (aHR = 2.06, 95% CI 1.85–2.29).

**Table 3:** Comparison of incidence and hazard ratio of depression stratified by sex, age and comorbidity between with and without Scrub typhus patients

Variable	Without scrub typhus				With scrub typhus				
	Event	Person-years	Rate^a^	Crude HR	Event	Person-years	Rate^a^	Crude HR (95% CI)	Adjusted HR^b^ (95% CI)
Sex									
Female	51	37,459	1.36	Reference	21	9232	2.27	1.67 (1.43, 1.95)*	1.68 (1.45, 1.96)*
Male	66	70,973	0.93	Reference	24	17,672	1.36	1.46 (1.29, 1.65)*	1.46 (1.29, 1.64)*
P for interaction									0.16
Stratify age									
≤ 49	52	65,025	0.80	Reference	25	16,123	1.55	1.94 (1.71, 2.19)*	1.95 (1.72, 2.20)*
50–64	35	28,372	1.23	Reference	15	7028	2.13	1.73 (1.46, 2.06)*	1.73 (1.46, 2.05)*
65 +	30	15,036	2.00	Reference	5	3753	1.33	0.67 (0.50, 1.02)	0.69 (0.52, 1.01)
P for interaction									< 0.001
Comorbidity^c^									
No	61	87,465	0.70	Reference	31	21,555	1.44	2.06 (1.85, 2.29)*	2.06 (1.85, 2.29)*
Yes	56	20,968	2.67	Reference	14	5350	2.62	0.98 (0.80, 1.20)	0.98 (0.80, 1.20)
P for interaction									< 0.001

^a^Incidence rate, per 1000 person-years; Crude HR, crude hazard ratio

^b^Multivariable analysis including age, and comorbidities of diabetes, hypertension, hyperlipidemia, CAD and stroke

^c^Patients with any one of the comorbidities diabetes, hypertension, hyperlipidemia, CAD and stroke were classified as the comorbidity group

* P < 0.05

## Discussion

ST is one of the most common mite-borne infectious diseases worldwide, and studies on the relationship between ST and depression are limited. This is the first study to report that ST patients have a 1.56-fold higher risk of depression than the general population.

### Role of vascular disease in depression and role of endothelial dysfunction in vascular disease and depression

In the past, depression was considered an acute and self-limiting illness related to the emotion of sadness. However, it is increasingly being considered a chronic disease. The main hypotheses for the etiology of depression include the monoamine hypothesis and the hypothalamic–pituitary–adrenal axis hypothesis [14]. The monoamine hypothesis implicates a deficiency of serotonin or norepinephrine neurotransmission in the brain [14], whereas the hypothalamic–pituitary–adrenal axis hypothesis implicates abnormalities in the cortisol response to stress [14]. Other possible pathophysiological mechanisms of depression include altered glutamatergic neurotransmission, reduced GABAergic neurotransmission, deficient neurosteroid synthesis, and impaired endogenous opioid function [15–18].

In addition, growing evidence suggests that vascular disease of the brain may predispose people to depression late in life [19]. The vascular depression hypothesis states that “cerebrovascular disease may predispose, precipitate, or perpetuate some geriatric depressive syndromes” [19]. Several types of studies, including clinical, neuroimaging, and neuropathologic studies, have investigated the association between vascular disease and depression. Many clinical studies have investigated the role of existing cardiovascular disease in increasing the risk of depression [20–22]. Vascular disease has a bidirectional relationship with depression: underlying cardiovascular disease can increase the risk of depression and a history of depression can increase the risk of cardiovascular disease [23]. This bidirectional relationship indicates that both depression and cardiovascular disease may share a common pathology. Numerous epidemiologic studies have associated a history of depression with subsequent development of ischemic heart disease [24–26]. Janusz reported that patients with depression had impaired arterial endothelial function [27]. Arterial endothelial dysfunction may be the common pathophysiology that induces both depression and cardiovascular disease. In neuroimaging studies, cerebral white matter lesions, thought to result from cerebrovascular brain damage [28], have been associated with depression [29, 30]. In addition, the presence of white matter hyperintensities and deep gray matter of the basal ganglia on T2-weighted or fluid-attenuated inversion recovery magnetic resonance imaging have been associated with vascular depression [31–34]. In a postmortem examination study, the rate of atheromatous disease was higher in patients with depression than in those without, with most of the difference resulting from increases in the cerebral vessels [35]. These findings suggest that vascular disease may be a predisposing factor for depression.

### Role of endothelial dysfunction in ST

Endothelial dysfunction is considered to be involved in the pathogenesis of ST [36]. In spotted fever group rickettsiae, rickettsiae attach and enter the host cell receptors by means of surface proteins ompB and ompA. These infections are vasculotrophic in nature, and current hypotheses regarding immunopathogenic mechanisms implicate oxidative stress, which leads to endothelial cell injury, increased microvascular permeability, and even the development of a hypercoagulant condition [36]. The endothelial dysfunction and systemic inflammation caused by ST may lead to subsequent development of cardiovascular disease [37] and depression.

### Other factors associated with depression

In the current study, we observed that compared with the non-ST group, the incidence of depression in the ST group was higher in patients of both sexes, in patients younger than 65 years, and in patients without comorbidities (namely diabetes, hypertension, hyperlipidemia, CAD, and stroke). This finding indicates that ST may be a risk factor for depression. As we know, ST is probably one of the most prevalent, under-recognized, under-diagnosed, and neglected but easily treatable disease in the world [38]. It occurs in rural residents and persons who engage in occupational or recreational behavior that brings them into contact with mite-infested habitats such as brush and grass [39]. People residing in rural areas, with occupational or recreational exposure to mite-infested habitats, may frequently suffer from ST infection with or without clear diagnoses. This kind of chronic exposure to ST may be the pathogenesis of developing subsequent depression.

The aHR of depression was 1.35-fold higher in women than in men (95% CI 1.24–1.47), which is consistent with the findings of a previous study [40]. In addition, the risk of depression was higher in patients with comorbidities, namely hypertension (aHR = 1.41, 95% CI 1.24–1.59), diabetes (aHR = 1.53, 95% CI 1.35–1.73), CAD (aHR = 1.51, 95% CI 1.29–1.76), hyperlipidemia (aHR = 2.08, 95% CI 1.81–2.40), and stroke (aHR = 1.52, 95% CI 1.27–1.81), suggesting that all comorbidities described in this study share at least some risk factors involved in the pathogenesis of depression, such as endothelial dysfunction. Interestingly, ST seems to increase the incidence of depression in the subgroups that we found to have a generally low risk of depression, such as people younger than 64 and people without comorbidities. In contrast, in the subgroups at high risk of developing depression, such as people older than 65 and people with comorbidities, ST did not increase the incidence of depression. These results suggest that patients older than 65, patients with comorbidities, and patients with ST may share risk factors involved in the pathogenesis of depression, such as endothelial dysfunction. The common risk factors involved in the pathogenesis of depression, such as endothelial dysfunction, may be enhanced in people older than 65 and those with comorbidities. Thus, the influence of ST infection is not obvious in these groups.

### Limitations

Owing to the mandatory nature of the NHI, we were able to enroll a large sample of patients in this study. However, our study has some limitations that must be addressed. First, this is not a prospective randomized controlled clinical trial, and confirming the association between ST and depression is difficult by employing a retrospective study design and the NHIRD. Therefore, a prospective randomized controlled study is recommended for clarifying the cause and effect relationship between ST and depression. Second, the diagnosis of ST was identified through the presence of ICD-9-CM codes; however, ST with presentation of mild symptoms might have been misclassified and coded as a flu-like disease, potentially leading to underestimation of the risk of depression. Third, our ST patients were hospitalized and might have had relatively severe symptoms. The higher risk of depression in our study might have been observed in only those ST patients whose symptoms were severe enough to require hospitalization. Finally, information on the daily physical activity, diet, socioeconomic status, family history, and drug regimens is not recorded in the NHIRD. These factors may influence the development of depression.

## Conclusions

In conclusion, this is the first study that investigated the relationship between ST and depression. Patients with ST have a 1.56-fold higher risk of depression than the general population. This finding highlights the role of endothelial dysfunction in depression. Additional studies should be conducted to clarify the cause and effect relationship between ST and depression. People with a history of ST infection should review its exposure risk factors to avoid chronic damage and any related diseases.

## Abbreviations

CAD: coronary artery disease
CI: confidence interval
HR: hazard ratio
ICD-9-CM: International Classification of Diseases, Ninth Revision, Clinical Modification
NHI: National Health Insurance
NHIRD: National Health Insurance Research Database
ST: scrub typhus
SD: standard deviation
